# Behavioral disinhibition in stroke

**DOI:** 10.3389/fneur.2024.1345756

**Published:** 2024-03-04

**Authors:** Wai Kwong Tang, Edward Hui, Thomas Wai Hong Leung

**Affiliations:** ^1^Department of Psychiatry, Chinese University of Hong Kong, Hong Kong, Hong Kong SAR, China; ^2^Department of Imaging and Interventional Radiology, Chinese University of Hong Kong, Hong Kong, Hong Kong SAR, China; ^3^Department of Medicine and Therapeutics, Chinese University of Hong Kong, Hong Kong, Hong Kong SAR, China

**Keywords:** stroke, behavior disinhibition, MRI, prefrontal cortex, anterior temporal lobe, caudate, thalamus

## Abstract

**Background:**

Post-stroke behavioral disinhibition (PSBD) is common in stroke survivors and often presents as impulsive, tactless or vulgar behavior. However, it often remains undiagnosed and thus untreated, even though it can lead to a longer length of stay in a rehabilitation facility. The proposed study will aim to evaluate the clinical, neuropsychological and magnetic resonance imaging (MRI) correlates of PSBD in a cohort of stroke survivors and describe its 12-month course.

**Methods:**

This prospective cohort study will recruit 237 patients and will be conducted at the Neurology Unit of the Prince of Wales Hospital. The project duration will be 24 months. The patients will be examined by multiple MRI methods, including diffusion-weighted imaging, within 1 week after stroke onset. The patients and their caregivers will receive a detailed assessment at a research clinic at 3, 9 and 15 months after stroke onset (T1, T2 and T3, respectively). The disinhibition subscale of the Frontal Systems Behavior Scale (FrSBe) will be completed by each subject and caregiver, and scores ≥65 will be considered to indicate PSBD.

A stepwise logistic regression will be performed to assess the importance of lesions in the regions of interest (ROIs), together with other significant variables identified in the univariate analyses. For patients with PSBD at T_1_, the FrSBe disinhibition scores will be compared between the groups of patients with and without ROI infarcts, using covariance analysis. The demographic, clinical and MRI variables of remitters and non-remitters will be examined again at T_2_ and T_3_ by logistic regression.

**Discussion:**

This project will be the first MRI study on PSBD in stroke survivors. The results will shed light on the associations of lesions in the orbitofrontal cortex, anterior temporal lobe and subcortical brain structures with the risk of PSBD. The obtained data will advance our understanding of the pathogenesis and clinical course of PSBD in stroke, as well as other neurological conditions. The findings are thus likely to be applicable to the large population of patients with neurological disorders at risk of PSBD and are expected to stimulate further research in this field.

## Background

For the purpose of this proposed study, behavioral disinhibition (BD) is defined as the inability to inhibit inappropriate behavior (1). Disinhibition interferes with the ability to inhibit automatic behavior, urges and emotions. It also impedes goal-directed behavior such as resisting temptation, delaying gratification and controlling impulses. Examples of BD include inappropriate comments, jokes, flamboyancy, lack of shame, impulsive behavior, disregard for conventions, poor risk assessment, undue familiarity, sexual acting out and vulgarity.

BD is a common phenomenon in cases of cerebral diseases such as frontal tumor (2), frontotemporal dementia (2–4), progressive supranuclear palsy (5, 6), amyotrophic lateral sclerosis (7, 8), multiple sclerosis (9), traumatic brain injury (1, 10, 11) and stroke (12, 13). For instance, the prevalence of BD in patients with frontotemporal dementia varies from 42 to 83% (3, 4, 14). BD is also common in patients with head injury, with a prevalence ranging from 19 to 32% (1, 10, 15). BD is associated with poor quality of life (7) and suicidality (16) in patients and with burden (7, 17, 18) and stress (19, 20) in their caregivers.

Post-stroke BD (PSBD) is common in stroke survivors and often presents as impulsive, tactless or vulgar behavior (21). Various studies have reported that 5 to 76% of stroke patients had PSBD at 4 days to 4 years post-stroke (12, 21–27). The frequency of PSBD, detected using the Neuropsychiatric Inventory (28), has been reported to range from 5 to 29% (12, 21, 24–26). Two local studies have found the frequency of PSBD to be 5 to 17% (25, 27). The clinical correlates of PSBD are unknown, while the correlates of BD in other neurological disorders have been suggested to include male sex (29, 30), severity of disease (20, 29), disability (10) and depressive and anxiety symptoms (9, 31).

The course of PSBD is uncertain. In a study of only 10 stroke survivors with PSBD, the remission rate at a 1-year follow-up was 90% (26). In contrast, another cross-sectional study of 274 stroke survivors reported the prevalence of PSBD as 22, 34 and 31% at 2.5 years, 2.5–5.5 years and beyond 5.5 years post-stroke, respectively, suggesting possible chronicity of PSBD (12). There is a lack of large-scale longitudinal studies on the course of PSBD. Similarly, the predictors of persistence of PSBD are unknown. Our previous research revealed the non-remission rate of post-stroke depression, another neuropsychiatric condition, at 1 year to be 66%, and the clinical correlates of persistence of post-stroke depression were found to be severity of depression, severity of stroke and cognitive functioning at baseline (32).

PSBD often remains undiagnosed and thus untreated, even though it can lead to a longer length of stay in a rehabilitation facility (26). Single case reports have suggested that transcranial direct current stimulation is useful in alleviating PSBD (13, 33). However, no high-quality trials on pharmacological and psychosocial treatments for PSBD have been conducted to date. Selective serotonin reuptake inhibitors (34, 35), bupropion (35), trazodone (36), aripiprazole (35), dextroamphetamine (35) and donepezil (37) may be useful pharmacological treatments for reducing BD in cases of neurological diseases, whereas cognitive and behavioral interventions may be useful non-pharmacological treatments (35, 38).

Starkstein and Robinson (39) suggested that most patients with BD have orbitofrontal cortex (OFC) and/or basotemporal dysfunction. Based on both contextual cues and object–reward associative memory, the OFC may promote or inhibit behavior that is programmed in the dorsal cortex (40). The basotemporal cortex and the OFC share prominent anatomical connections that could underlie the association between frontal lobe-related volitional and psychomotor behavior and limbic system-related emotional drive. Thus, dysfunction of this system may result in motor disinhibition, instinctive disinhibition and emotional disinhibition. Tekin and Cummings (41) proposed that BD occurs due to dysfunction of the orbitofrontal subcortical circuit (OFSC). The principal components of this brain circuit are the medial OFC, frontal subcortical white matter, caudate and thalamus. The relationships between BD and dysfunctions in the main components of this circuit are discussed in the following paragraphs.

BD is common in patients with frontal lobe pathologies (42) such as frontal tumors (2), frontotemporal dementia (4, 43) and frontal injuries (1). Frontal lobe stroke is associated with reduced emotional intelligence (44). BD is a common sequela of frontal lobe tumors and is related to lesions in the OFC in patients with traumatic brain injuries (1, 45). In patients with mild cognitive impairment, dementia or frontotemporal dementia, BD is positively correlated with atrophy (31, 46, 47) and hypometabolism in the OFC (48). Inhibitory dysfunction in patients with Parkinson’s disease is also related to OFC atrophy (49). Our team previously demonstrated the association of frontal infarcts with another post-stroke neuropsychiatric condition, namely anxiety (50).

In addition to the frontal cortex, temporal lobe structures have been implicated in BD. Socially appropriate behavior requires knowledge of adequate social actions within a given sequential context. Such social knowledge or concepts or emotions are represented in the anterior temporal lobe (ATL) (51, 52). There is strong evidence that the ATL is involved in inappropriate social behavior (53, 54). For example, BD symptoms were found to be present in 65% of patients with temporal lobe atrophy (55). BD in patients with temporal-variant frontotemporal dementia has been found to be related to temporal atrophy (56, 57). Zahn et al. (51) reported that patients with frontotemporal lobar degeneration and corticobasal syndrome accompanied by ATL degeneration had significantly more impairment in social concepts and showed more BD symptoms than those without ATL degeneration. In a lesion–symptom mapping study of patients with traumatic brain injuries, damage to the right temporal lobe, including the pole, was associated with greater BD symptoms (1).

BD has been linked to thalamic and caudate lesions and is thought to arise due to the interruption of the prefrontal–subcortical network (58). PSBD has been linked to paramedian thalamic infarction (59–61). BD is also a known feature of basal ganglia disorders (61–65). Mendez et al. (66) attributed BD to ventromedial caudate lesions. Case reports have linked BD to caudate infarction (64, 67). BD is present in 9 to 20% of patients with Parkinson’s disease (19, 63, 68) and 11% of patients with caudate lesions (62). Reduced gray matter density and altered metabolic connectivity in the striatum have also been associated with BD in patients with frontotemporal dementia (62, 69). Our team found that caudate infarcts are linked to a post-stroke neuropsychiatric condition, namely fatigue (70).

Very few structural brain imaging studies have been published on PSBD (12, 22, 58, 59, 71). Single case reports and case series have linked PSBD to paramedian thalamic (58, 59), caudate (55), supratentorial (22) and subtentorial (71) infarcts. Van Almenkerk et al. (12) reported no association between the prevalence of PSBD and the laterality of stroke. An increase in BD symptoms was noted in 79 patients with subtentorial infarcts compared with 10 patients with parietal/occipital infarcts (71). The limitations of these studies include mixed cohorts of acute and chronic stroke survivors (12) and a lack of detailed radiological examination. Furthermore, the classification of infarct locations was rather crude, namely subtentorial versus parietal/occipital or supratentorial (22, 71), left versus right (12, 22), cortical versus subcortical and anterior versus posterior (22). This proposed project will be the first magnetic resonance imaging (MRI) study on PSBD in stroke survivors. The results of our investigation of the associations between lesions in the OFC, ATL and subcortical structures and the risk of PSBD can advance our understanding of the pathogenesis and clinical course of PSBD in stroke as well as other neurological conditions.

### Aims and hypotheses to be tested

The main objective of the proposed study will be to evaluate the clinical and MRI correlates and the 12-month course of PSBD in a cohort of stroke survivors. The regions of interest (ROIs) will be the OFC, ATL, thalamus, and caudate. In addition to individual brain regions, the presence of infarcts affecting structures of the OFSC will be evaluated. The occipital and parietal lobes will be included as control regions.

#### Hypotheses

Four hypotheses will be tested: (i) patients with PSBD have more infarcts in the ROIs, but not in the control regions, than those without PSBD; (ii) there is a significant positive correlation between the number of infarcts in the ROIs and the severity of PSBD; (iii) 66% (32) of patients with PSBD at baseline continue to have PSBD 12 months after the first assessment; and (iv) the severity of PSBD, severity of stroke and level of cognitive functioning at baseline predict the persistence of PSBD (32).

## Methods

### Recruitment of subjects

**Figure 1:**
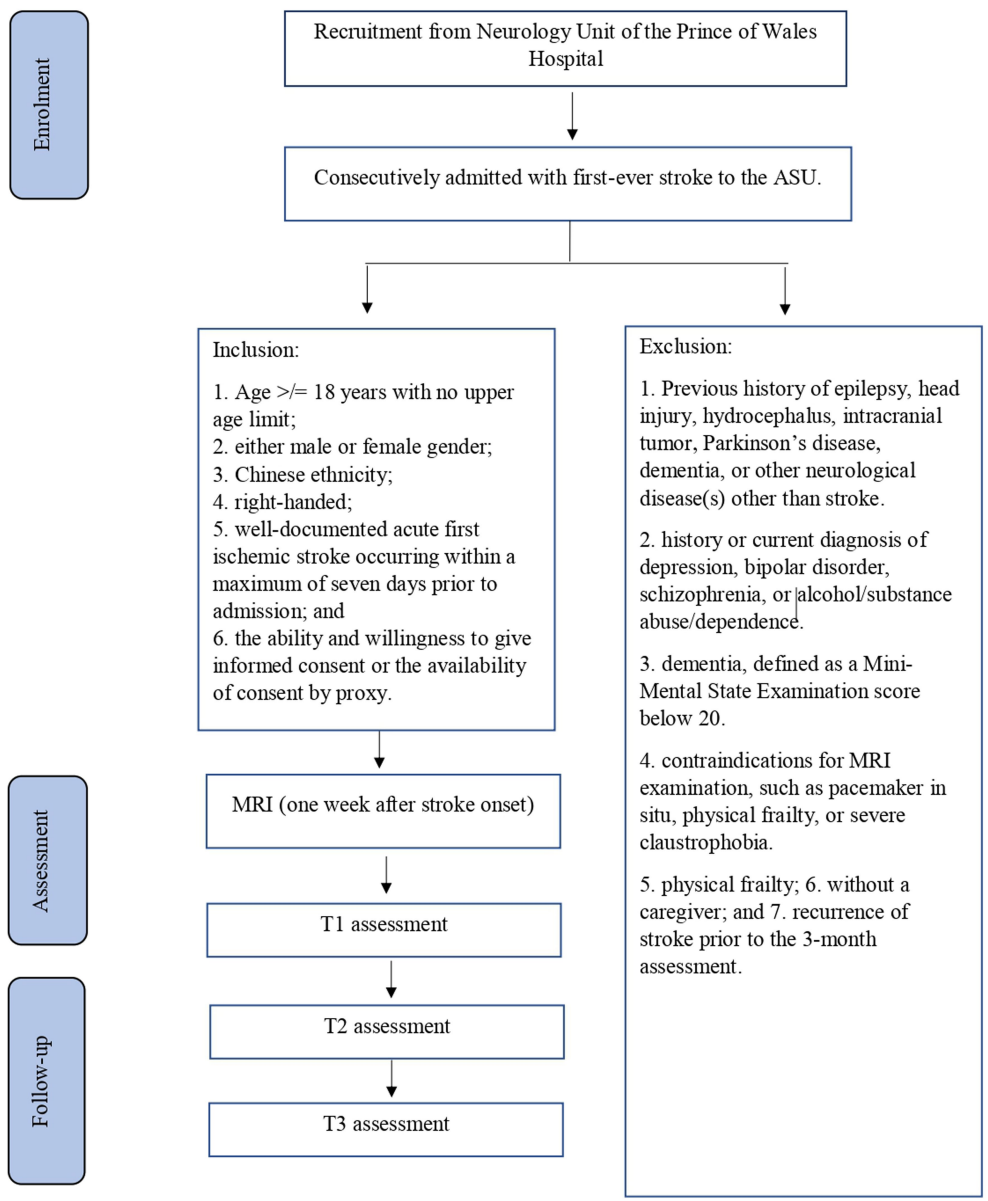
Details of recruitment.

The planned study will be a prospective cohort study of stroke survivors. Details of recruitment are shown in Figure 1. Subjects will be recruited from among patients with first-ever stroke who are consecutively admitted to the Acute Stroke Unit (ASU) of the Prince of Wales Hospital (PWH). The PWH is a general hospital serving a population of 800,000 in Hong Kong. The ASU treats approximately 93% of all acute stroke patients admitted to the PWH, with the majority of the remaining 7% admitted to the neurosurgery unit. All of the acute stroke patients (*n* = 500) consecutively admitted to the ASU over a 12-month period will be invited to participate in the study. A research assistant (RA) will visit the ASU daily to identify eligible patients and obtain their written consent for inclusion in the study. It is estimated that approximately 80% of these 500 patients (*n* = 500 × 80% = 400) will have ischemic stroke and that MRI examination will be contraindicated in 10% of them, leaving 360 potential subjects (400 × 90%). According to our previous findings (72), the mortality rate at 3 months post-stroke is around 12%; thus, 316 [360 × (100% − 12%)] potential subjects will be approached. Of these survivors, 25% will not meet the inclusion criteria (72). Hence, the number of possible subjects will be around 237 [316 × (100% − 25%)] (72)^.^ Assuming a dropout rate of 20%, 190 [237 × (100–20%)] patients are expected to complete the 12-month follow-up assessment.

### Sample size estimation

Two hundred and thirty-seven patients will be recruited. If no significant correlations between BD and lesion site are found in a sample of this size, it is unlikely that any clinically meaningful effects of lesions would be found with a larger sample. As there are no published data on the location of infarcts in patients with PSBD, we calculated the sample size using the figures reported for another neuropsychiatric disorder in stroke. In a report on anxiety in patients with stroke, 21.4% of those with anxiety had frontal infarcts versus only 8.6% of patients without anxiety, and the odds ratio was 2.9 (50). Using these figures as the estimate, a sample size of 237 will have 94.7% power in identifying frontal infarcts as a predictor of PSBD in stroke, using a multivariate logistic regression analysis (73). Give that 190 patients are expected to complete the 12-month assessment, this will provide at least 88% power in identifying the severity of PSBD, severity of stroke and cognitive functioning at baseline as predictors of the persistence of PSBD, using a two-sample t-test (32).

### Eligibility criteria

#### Inclusion and exclusion criteria

The following inclusion criteria will be applied: (i) Age ≥ 18 years with no upper age limit; (ii) either male or female gender; (iii) well-documented acute first ischemic stroke that has occurred within a maximum of 7 days prior to admission; and (iv) the ability and willingness to give informed consent or the availability of consent by proxy, obtained from patients’ next of kin.

The following exclusion criteria will be applied: (i) A history of epilepsy, head injury, hydrocephalus, intracranial tumor, Parkinson’s disease, dementia or neurological disease (s) other than stroke; (ii) history or current diagnosis of depression, bipolar disorder, schizophrenia or alcohol/substance abuse/dependence; (iii) dementia, defined as a Mini-Mental State Examination score below 20; (iv) contraindications for MRI examination, such as a pacemaker *in situ*, physical frailty or severe claustrophobia; and (v) recurrence of stroke prior to the 3-month assessment.

### Data collection

Details of the data collection schedule are shown in Appendix Table 1. Written or proxy consent will be obtained from all of the patients. The number of patients excluded and reasons for exclusion will be recorded. The following demographic, psychosocial and medical data will be collected from all subjects: age, sex, education and date of stroke onset. Subjects’ clinical data and information on neurological impairments, including aphasia and dysarthria measured using the National Institute of Health Stroke Scale (74) (NIHSS), will be extracted from the Stroke Registry, which is maintained by a full-time, trained research nurse.

### Assessment of PSBD

Three months after the onset of the index stroke (T1), the patients and their caregivers will receive the following assessments at a research clinic. The timing of the assessment is consistent with other studies of PSBD (24, 27). A psychiatrist blind to the subjects’ radiological data will conduct a clinical interview at the research clinic. PSBD will be assessed using the disinhibition subscale of the validated Frontal Systems Behavior Scale (FrSBe). The FrSBe is a 46-item questionnaire that assesses three frontal behavioral domains: apathy, disinhibition and executive dysfunction. The disinhibition subscale contains 15 items that assess problems with inhibitory control of actions and emotions, including impulsivity, hyperactivity, social inappropriateness, emotional lability, explosiveness and irritability. All items are rated on a 5-point Likert scale: 1 (almost never), 2 (seldom), 3 (sometimes), 4 (frequently), 5 (almost always). Raw scores are converted to normative T-scores (sex-, age-and education-matched) for each behavior, and an overall “frontal dysfunction” score is also calculated. Higher scores indicate greater dysfunction. Scores ≥65 are considered clinically significant, 60–64 are considered borderline and < 60 are considered normal (75, 76). The FrSBe has a clear three-factor structure, and the corresponding subscales have previously shown good validity and reliability. The disinhibition subscale has high internal consistency, indicated by a Cronbach’s alpha coefficient of 0.89 (77). The FrSBe has been used for the assessment of frontal system dysfunction in stroke survivors (44, 78, 79).

A trained RA, blind to the subjects’ radiological data, will measure the level of physical functioning, depressive and anxiety symptoms, cognitive functioning, social functioning, quality of life and anosognosia using the Barthel Index (80) (BI), the Beck Depression Inventory (81) (BDI), the anxiety subscale of the Hospital Anxiety Depression Scale (82) (HADSA), the Montreal Cognitive Assessment (MoCA) (83), the Computerized Adaptive Test of Social Functioning (Social-CAT) (84), the Stroke-Specific Quality of Life Scale (SSQoL) (85) and the Self-Awareness of Deficits Interview (SADI) (86), respectively. Proxy ratings by the caregivers will be obtained for subjects with marked aphasia.

Follow-up assessments of PSBD will be conducted for all subjects at 9 months (T2) and 15 months (T3) post-stroke. All of the instruments (FrSBe, BI, MoCA, BDI, HADSA, Social-CAT and SSQoL) will be repeated during the follow-up assessments (Appendix Table 1).

### MRI examination and analysis

Patients will be examined by MRI within 1 week after stroke onset. All scans will be performed using a 3 T scanner (Philips Achieva 3.0 T, X Series, Quasar Dual MRI System) with standardized sequences, including diffusion-weighted imaging (DWI), 3D T1-weighted, T2-weighted, fluid-attenuated inversion recovery (FLAIR) and susceptibility-weighted imaging (SWI). An experienced neuroradiologist blind to the subjects’ PSBD status will assess the MRI images. Acute infarct will be defined as a hyperintense lesion on DWI with corresponding hypointensity on the apparent diffusion coefficient map. White matter hyperintensities (WMH) will be defined as hyperintensities ≥5 mm that are ill-defined on FLAIR images but are isointense with normal brain parenchyma on T1-weighted images. Lesions equivalent to the signal characteristics of cerebrospinal fluid on T1-weighted images and measuring more than 3 mm in diameter, as well as wedge-shaped cortico-subcortical lesions, will be regarded as old/lacunar infarcts. Microbleeds will be defined as dot-like hypointensities on SWI. The total number of microbleeds will be determined. The number of microbleeds in the basal ganglia and thalamus will also be noted separately. All raw data will be transferred to the PALS system (Carestream Solutions).

An ordinal scale devised and validated by Staals et al. (87) will be used to estimate the total small vessel disease (SVD) burden. Briefly, the presence of each of the four MRI markers for SVD (WMHs, lacunae, cerebral microbleeds and perivascular spaces) will be summed to form a total SVD score ranging from 0 to 4. WMHs will be assessed using the Fazekas scale, with scores ranging from 0 to 3. Extensive WMH will be indicated by deep WMHs that score 2 or 3 or by periventricular WMHs that score 3. One point will be given for any extensive WMH, cerebral microbleed or lacuna. One point will be awarded to perivascular spaces when more than 10 are located on one side of a single slice in the basal ganglia.

#### MRI pre-processing

This will include non-uniformity correction (88), spatial standardization and brain extraction (excluding the skull). To ensure that the brain structure volumes are comparable among subjects, the MRI data of each subject will be transformed from its original space to a common stereotactic space using multi-scale affine registration (89). Brain regions will be automatically segmented from the head MRI data using the brain extraction tool (90).

#### Brain segmentation

Brain tissue will be classified into gray matter, white matter and cerebrospinal fluid (91). Whole-brain segmentation will be achieved using an atlas-based approach (92), which automatically adjusts the existing atlas intensity model to newly inputted data. The ROIs and other brain regions will be segmented and their volumes quantified using the Tamarac brain atlas (93) and demon registration (94).

#### Infarct segmentation and quantification

Infarcts will be delineated semi-automatically as high-intensity regions on diffusion-weighted images and WMHs as high-density regions on FLAIR images (and isointense on T1-weighted images) using ITK-SNAP software. The segmented infarct and WMH regions will be combined with the ROI and other brain-region masks generated in the previous step. The infarct and WMH pixels that fall within the ROIs and other brain regions will then be calculated.

### Statistical analysis

All of the variables will be tested for normality using Kolmogorov–Smirnov tests with a significance threshold of *p* < 0.05. Demographic, clinical and MRI variables (age; gender; NIHSS, BI, HADSA, Social-CAT, SSQoL, BDI and MoCA scores; ROI and OFSC infracts; microbleeds; WMH volumes; and total SVD scores) will be compared between patients with and without PSBD at T_1_ using the χ^2^ test, Student’s t-test or the Mann–Whitney U test, as appropriate. Stepwise logistic regression will be performed to assess the importance of lesions in the ROIs, together with other significant variables identified in the above univariate analyses. For patients with PSBD at T_1_, the FrSBe disinhibition scores for the groups with and without ROI infarcts will be compared using covariance analysis. The demographic, clinical and MRI variables of remitters and non-remitters at T_2_ and T_3_ will be examined again using logistic regression. We will also test a series of generalized estimating equation models to evaluate the association between the clinical and brain MRI characteristics and risk of PSBD across all follow-up assessments (T_1_, T_2_ and T_3_). First, we will run a univariate model to fit a logistic regression. Next, we will examine the association between the demographic variables and concurrent medical diseases and the risk of PSBD. The second model will comprise baseline FrSBe disinhibition, NIHSS and MoCA scores added to the previous model. The brain MRI characteristics will be entered in the final model. The level of significance will be set at 0.05.In addition to the above pre-planned analysis, an exploratory voxel-based analysis will be performed.

## Discussion

We will try to achieve a homogeneous patient population by narrowing the criteria of age and duration of PSBD. Patients with other causes of PSBD, such as psychiatric or neurological disorders, will be excluded. This project will be the first longitudinal study to examine the role of the OFC, ATL, caudate and thalamus in a large sample of consecutively admitted stroke survivors with PSBD. The results will shed light on the association between the above brain regions and PSBD. They are thus likely to be applicable to the large population of patients with neurological disorders at risk of BD and should also stimulate further research in this field.

## Ethics statement

The studies involving humans were approved by Joint Chinese University of Hong Kong–New Territories East Cluster Clinical Research Ethics Committee. The studies were conducted in accordance with the local legislation and institutional requirements. The participants provided their written informed consent to participate in this study.

## Author contributions

WKT: Conceptualization, Methodology, Writing – original draft, Writing – review & editing. EH: Conceptualization, Methodology, Writing – review & editing. TWHL: Conceptualization, Methodology, Writing – review & editing.

## Appendix

TABLE 1 Data collection schedule.

**Table 1:**

Study period	T0	T1	T2	T3
Timepoints (months post-stroke onset)	0	3	9	15
Visits	0	1	2	3
MRI	X			
Review of inclusion/exclusion criteria	X			
Informed consent	X			
FrSBe, BI, MoCA, BDI, HADSA, Social-CAT, SSQoL		X	X	X
